# Studies on the Two Thymine Residues in the Catalytic Core of 10-23 DNAzyme: The Impact on the Catalysis of Their 5-Substituted Functional Groups

**DOI:** 10.3390/molecules22071011

**Published:** 2017-06-22

**Authors:** Pengyu Li, Shanshan Du, Yang Li, Junlin He

**Affiliations:** 1 College of Life Sciences, Guizhou University, Guiyang 550025, China; lipengyu1@126.com (P.L.); shanshandu1992@163.com (S.D.); liyang920312@163.com (Y.L.); 2 Laboratory of Bioorganic chemistry, Beijing Institute of Pharmacology and Toxicology, Beijing 100850, China

**Keywords:** 10-23 DNAzyme, chemical modifications, thymine, functional group, catalytic core, observed catalytic constants

## Abstract

In the 15-mer catalytic core of 10-23 DNAzyme, each residue contributes to the catalytic conformation differently. Here, the critically conserved T4 and the least conserved T8 were modified on their 5-position with hydroxyl, imidazolyl, and amino groups with a hydrogen-bonding ability. These external functional groups induced new interactions within the catalytic core, resulting in both negative and positive effects on the catalytic activity of 10-23 DNAzyme, and the different linkages could be used to modulate the effect of the functional groups. The conservation of T4 and T8 could be recognized at the level of the nucleobase, but at the level of the functional group, T4 is not completely conserved. Their 5-methyl groups could be modified for a better performance in terms of the DNAzyme.

## 1. Introduction

10-23 DNAzyme is a small catalytic DNA molecule selected from artificial in vitro selection [1]. Its catalytic cleaving ability on complementary RNA has attracted much attention, from practical therapeutics [2,3,4] to catalytic mechanism studies [5,6]. Furthermore, it is an ideal model for studying the artificial functions and related tertiary structures of DNA molecules of non-genetic carriers, especially when no counterpart of DNAzyme has yet been found in nature. The composition of the secondary motifs (the duplex-loop-duplex) seems to be clear and simple, but no profile has been generated for the tertiary structure and the critical catalytic conformation, in which the specific catalytic groups conduct the cleavage reaction with the help of Mg^2+^. Screening approaches have been used for the nucleobase [7,8,9], sugar-phosphate moiety [10], and non-bridging oxygen atom of the phosphate group [11]. It could not be concluded that any residues or functional groups are directly involved in the catalytic reaction, but the importance of each nucleotide has been recognized by these screening approaches. Among the 15 residues in the catalytic core, T8 was suggested to be the least conserved residue, which is in contrast to the other highly conserved T4 residue, indicating their unique position-dependent contributions to the catalytic activity.

Considering the completely different conservation of T4 and T8, several chemical modifications have been conducted to gain an insight into the structural basis. When phosphorothioate linkage and 2’-*O*-methyl were applied to T8, the two most frequently used chemical modifications for providing the resistance of oligonucleotides against nucleases, no significant negative effect on the cleavage activity of the DNAzyme was observed [12,13]. In contrast, T4 is always negatively affected by these modifications. But, it is noteworthy that both T4 and T8 are sensitive to its sugar conformation, as demonstrated by (*R*)- and (*S*)-2’-C-methyl-thymidine and LNA [14,15]. The nucleobase thymine was also demonstrated to be sensitive to the modifications. A large caging group (6-nitropiperonyloxymethyl, NPOM) at N-1 of thymine at T4 could abrogate the catalytic activity completely, while partial activity remained with the caged thymine of T8 [16]. The 5-substituted aromatic group of thymine could produce a favorable or unfavorable effect at T4 and T8, depending on its geometric configuration [17]. The two thymine residues of T4 and T8 were further modified with imidazolyl and ester groups, and the extra functional groups at T8 were found to be helpful for the catalytic reaction, even in the absence of Mg^2+^ [18]. When T4 was conducted for these modifications, a negative effect was mostly observed, in agreement with its critical conservation.

**Scheme 1:**
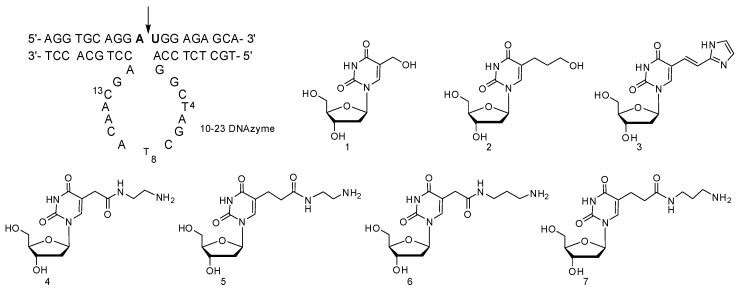
10-23 DNAzyme targeting against VEGFR2 mRNA and the 2’-deoxythymidine analogues for the modifications on T4 and T8 at the catalytic core. The bold letter represents the RNA residues in the substrate to be cleaved, as the arrow indicates.

These studies demonstrated that the effect of T4 and T8 can be modulated by changing the interactions around the two thymine residues. Here, we report new modifications on the two thymine residues with the hydroxyl group (in compounds **1** and **2**), imidazolyl group (in compound **3**), and amino group (in compounds **4**–**7**) at the 5-position by different linkages (Scheme 1). More hydrogen-bonding interactions around T4 and T8 were expected to have an effect on the catalytic reaction of 10-23 DNAzyme (Scheme 1) against VEGFR 2 mRNA [19], and it was hoped that a chemical modification approach for a positive effect was found.

## 2. Results and Discussion

### 2.1. Nucleoside Analogues

**Scheme 2:**
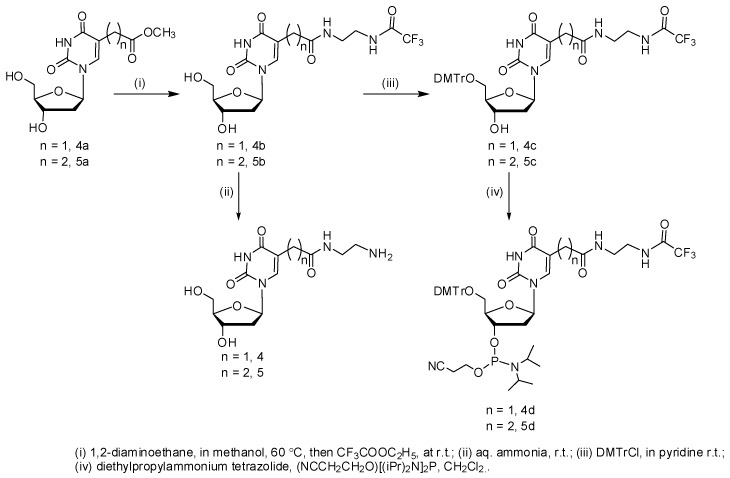
Synthesis of compounds **4** and **5** and the phosphoramidites **4d** and **5d**.

**Scheme 3:**
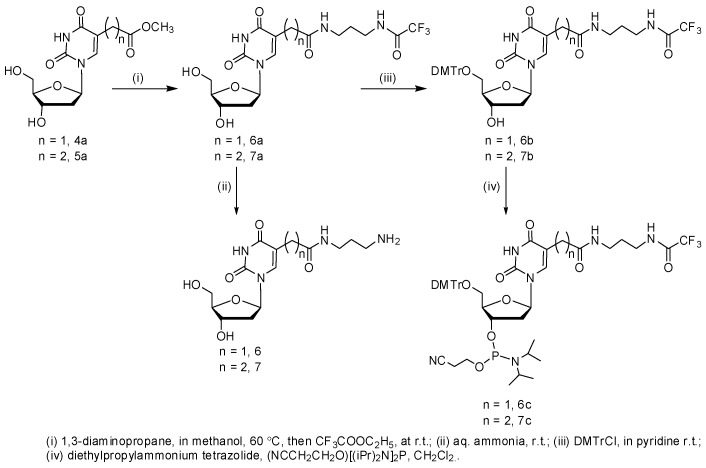
Synthesis of compounds **6** and **7** and the phosphoramidites **6c** and **7c**.

From chemical modifications on the thymine base and sugar-phosphate moieties of T4 and T8, its functional groups and the aromatic plane are supposed to form hydrogen-bonding and stacking interactions with other residues in the catalytic loop, and these interactions can be modulated to exert more influence on the catalytic conformation. Here, chemical modifications were only conducted at its 5-position, and the functional groups of its hydrogen-bonding ability were introduced. The hydroxyl group was linked by an alkyl bridge in compounds **1** and **2**, the imidazolyl group was linked by a vinylene group in compound **3**, and the amino group was linked through an amido group in compounds **4**–**7** of different lengths. Compounds **1**–**3** and their phosphoramidites were synthesized according to the literature [20,21]. Compounds **4** to **7** and their phosphoramidites were synthesized as shown in Scheme 2 and Scheme 3. 5-methoxycarbonylmethyl-2’-deoxyuridine (**4a**) and 5-methoxycarbonylethyl-2’-deoxyuridine (**5a**) were prepared from the glycosylation of 5-methoxycarbonylmethyl-uracil [22]. With the ester-amide exchange reaction with 1,2-diaminoethane, the 5-carbonylmethyl ester of compound **4a** was converted to 5-[*N*-(2-aminoethyl)carbamoylmethyl] of compound **4b** and 5-[*N*-(3-aminoethyl)carbamoylethyl] of compound **5b**; employing the same reaction with 1,3-diaminopropane, **4a** and **5a** were converted to the compounds **6a** and **7a**, respectively. The 5-substituted terminal amino group was protected with the trifluoroacetyl group [23]. The tritylation of their 5’-OH offered the corresponding compounds **4c** and **5c**, as well as **6b** and **7b**. Further conversion to the phosphoramidites was conducted to obtain the phosphoroamidites **4d**, **5d**, **6c**, and **7c**, for the solid-phase synthesis of the DNAzymes. Compounds **4b**, **5a**, **6a**, and **7a** could be deprotected to obtain the corresponding nucleosides **4**, **5**, **6**, and **7**. In these four compounds, the 5-positioned amino group was linked with a different linkage length and the amido group, in order to produce different effects on the catalytic ability of the DNAzyme.

### 2.2. Oligonucleotides

The solid-phase synthesis was conducted on an ABI 392, with a 1 µmol scale. The coupling time of the phosphoramidites of compounds **1**–**7** was extended to 3 min. The deprotection of the oligodeoxynucleotides was conducted with conc. aq. ammonia at 55 °C for 16 h. For the oligonucleotides containing compounds **1** and **2**, further cleavage of the tert-butyldiphenylsilyl group of the 5-substituted hydroxyl group was conducted with 1 M TBAF/THF. All of the deprotected oligonucleotides were purified with 20% denaturing PAGE (8 M urea). Desaltation was conducted with a Sep-Pak column and washed with sterilized bidistilled water, and the DNAzyme was eluted with 70% methanol/water (*v*/*v*) and lyophilized before being stored at −30 °C. The sequences of modified DNAzymes and the characterization results are listed in Table 1 (mass spectrum in Supplementary materials).

### 2.3. The Effect of the Modified Catalytic Loop on the DNAzyme-Substrate Complexes

In the DNAzyme-substrate complex, the duplex-loop-duplex contributes to a stable and specific tertiary structure for the catalytic reaction. Mg^2+^ was suggested to be delicately complexed for stabilizing and catalyzing roles. Therefore, the influence of the modified loop on the complex formation was studied with thermal stability (T_m_) and circular dichroism (CD).

**Figure 1:**
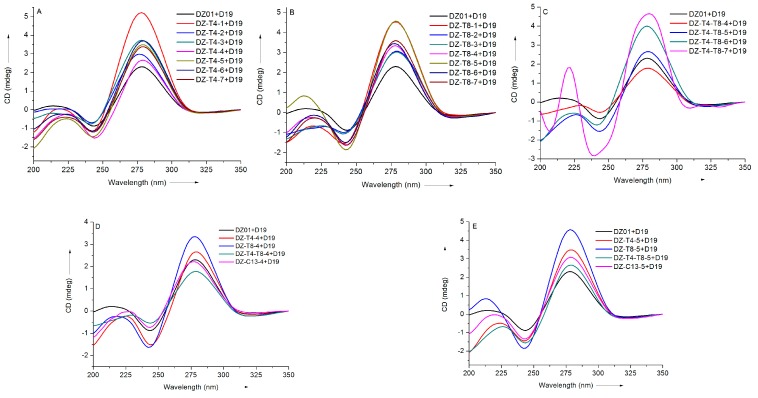
CD spectra of the DNAzyme-substrate complexes in the reaction conditions. Comparisons of the CD spectra were made between the modified DNAzyme-substrate complexes with different nucleoside analogues and at different positions: (**A**) Modified complexes at T4 with nucleoside analogues **1**–**7**; (**B**) Modified complexes at T8 with nucleoside analogues **1**–**7**; (**C**) Modified complexes at T4 and T8 with nucleoside analogues **4**–**7**; (**D**) Modified complexes with compound **4** at different positions; (**E**) Modified complexes with compound **5** at different positions.

The DNAzyme-substrate complex was measured under the reaction conditions (50 mM Tris-HCl, pH 7.5, 2 mM Mg^2+^), and the full-DNA substrate D19 was used instead of the chimeric substrate, to avoid the cleavage of the substrate in the reaction buffer. From the measured T_m_ values (Table 2), it seems that there was no significant difference between the stacking strength of the nucleobases in the catalytic loop. The thermal stabilities ensured that the modified DNAzyme could bind with the substrate, as the first step of the catalytic reaction. On the other hand, the changes of CD spectra shown in Figure 1 could imply conformational differences related to the unmodified 10-23 DNAzyme (**DZ01**). The 5-positioned external functional groups produced new interactions with other residues in the catalytic core and exerted some influence on the stacking mode of the two thymine residues, leading to conformational changes. The conformational changes are different from each other, depending on the properties of different functional groups and their positions in the catalytic core.

### 2.4. The Catalytic Activities of the Modified DNAzymes

**Figure 2:**
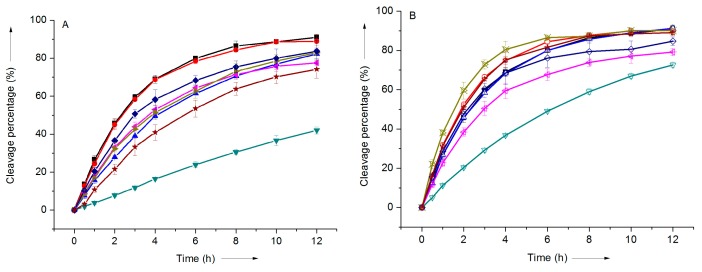
The cleavage reaction profiles of DNAzymes under single turnover conditions. (**A**) The DNAzymes modified at T4 were compared; (**B**) The DNAzymes modified at T8 were compared. Each DNAzyme was specified: **DZ01** (solid square, black line), **DZ-T4-1**(solid circle, red line), **DZ-T4-2** (solid triangle, blue line), **DZ-T4-3** (solid downtriangle, dark cyan line), **DZ-T4-4** (solid left triangle, magenta line), **DZ-T4-5** (solid right triangle, dark yellow line), **DZ-T4-6** (solid diamond, navy line), **DZ-T4-7** (solid star, mine line), **DZ-T8-1** (empty circle, red line), **DZ-T8-2** (empty triangle, blue line), **DZ-T8-3** (empty down triangle, dark cyan line), **DZ-T8-4** (empty left triangle, magenta line), **DZ-T8-5** (empty right triangle, dark yellow line), **DZ-T8-6** (empty diamond, navy line), **DZ-T8-7** (empty star, wine line).

The catalytic activities of the modified DNAzymes were evaluated under single-turnover conditions against the chimeric substrate D19AU, with rAU as the cleavage site. No reaction was observed without Mg^2+^, indicating that all of the extra functional groups could not behave like Mg^2+^. The cleavage profiles of the DNAzymes are shown in Figure 2.

With compound **1**, the external hydroxyl group was introduced to the catalytic loop through substitution at T4 (**DZ-T4-1**) or T8 (**DZ-T8-1**), and the observed rate constants indicated that the 5-substituted hydroxyl group at T8 could produce a slightly favorable effect on the cleavage reaction (Table 3), while no influence was observed for T4 substitution, which is in contrast with the drastic negative effect of most modifications at T4. The interaction related to this extra hydroxyl group was then studied with compound **2**. The decreased *k*_obs_ of **DZ-T4-2** indicated that the one-bond-longer linkage permitted the hydroxyl group to induce different interactions, leading to an unfavorable effect at T4. T8 was less affected by such a structural change (**DZ-T8-2**).

It has been reported that the contribution of T8 could be modulated by changing its stacking interaction with 5-substituted azobenzene groups, where the specific stacking of the aromatic group with a different configuration could induce a favorable or unfavorable effect for the contribution of T8 [17]. Compound **3** was used to introduce a rigidly conjugated imidazolyl group for the enlarged base stacking and hydrogen-bonding interactions. The obvious decrease in the observed rate constants of **DZ-T4-3** and **DZ-T8-3** indicated that these new interactions induced an unfavorable conformational change of the catalytic loop. It seems that the original base stacking interaction of T4 and T8 is very important, even for the least conserved T8. It was also convinced by the sugar modification of a different configuration at T4 and T8, where the (*R*)- and (*S*)-2’-*C*-methyl-thymidine and the locked conformation with LNA could lead to the different spatial occupation of thymine compared to the 2’-deoxyribose moiety, with an unfavorable impact on the catalytic activity [14,15].

With compounds **4**–**7**, an amino group was introduced, together with the amido linkage at the 5-position of thymine. As shown in Table 3, T4 is less compatible with these changes than T8. However, the partial retention of the catalytic activity demonstrated that the conservation of T4 could be recognized at the level of the thymine base, because any replacement of this thymine with other nucleobases led to an almost complete loss of activity [8]. On the other hand, although it has been demonstrated that several modifications led to a drastic loss of activity [15,18], T4 is not critically conserved at the 5-methyl group. This 5-methyl group could be modified for a better performance of T4 with a delicately designed substituent.

At the least conserved T8, the influence of the new substituents from compounds **4**–**7** was not always positive. The importance of the linkage was evident for the effect of the substituent. The decreased *k*_obs_ of **DZ-T8-4** indicated that the new interactions related to the external amino and amido groups induced an unfavorable effect on the catalytic conformation, but only one-carbon-longer change in **DZ-T8-5** led to a new positive effect. In addition, from the different activities between **DZ-T8-5** and **DZ-T8-6** with the same length of the linkage, the amido group in the linkage seems to be involved in the interactions of the new residues **5** and **6** at T8. Further extension of the terminal amino group could not induce a more positive effect in **DZ-T8-7**. These results indicated that T8 is also very sensitive to the chemical modifications at its 5-position.

Combinational modifications on both T4 and T8 with the same compound were conducted and different combinatorial effects were observed for each of the four modified residues compared to the corresponding single substitutions. The negative effect from the substitutions at T4 was always compensated by the further substitution at T8, by which a favorable conformational change was supposed to be driven. Especially in the case of compound **4**, its negative effect at T4 or T8 was deleted by the combined substitutions. These results further indicated the delicate influence of the 5-substituents of T4 and T8. On the other hand, these results could not imply that the modified residues at T4 and T8 are directly related to each other, based on the completely different conservation of T4 and T8. In the catalytic core of 10-23 DNAzyme, each residue was suggested to have its own interaction network with nearby residues, contributing to the catalytic conformation in its own way. Therefore, the observed rate constants of the modified DNAzymes reflected the comprehensive result of complex interactions between the external functional groups with the internal residues in the catalytic loop, and the additive effect could not be expected, depending on the specific residues and the modifications.

At C13, its 4-amino group has been demonstrated to be very important for its contribution, and partial activity could be retained by the replacement of adenine, while thymine could not be used for this position [8]. Here, the amino-modified thymine analogues **4**–**7** at C13 led to the complete loss of catalytic activity of the DNAzyme, which meant that the interactions derived from C13 could be very specific for the right catalytic conformation, including its base-stacking mode and the original functional groups for other interactions. 

In previous studies on the conservation of residues, the uniqueness of each residue in the catalytic core of 10-23 DNAzyme has been demonstrated by the inter-replacement of the canonical residues [7]. Therefore, the design of nucleoside analogues for the corresponding residues/nucleosides has been the first consideration in our approach for the chemical modification of the catalytic core. With the modification at the level of functional groups, the most conserved guanine residues [24] and T4 could be modified for a better performance. On the other hand, the selection of functional groups and the linkage is very important for the catalytic activity, as reported for the modifications of the 6-amino group of adenine [25,18]. These results demonstrated that the catalytic conformation of 10-23 DNAzyme is very sensitive to any structural changes, but it could be optimized.

## 3. Materials and Methods

### 3.1. General

Commercially available chemicals were used without purification. Dichloromethane was redistilled and dried on anhydr. K_2_CO_3_. 1,2-Diaminoethane and 1,3-diaminopropane were redistilled before use. Silica gel for TLC and flash column chromatography was obtained from Qingdao Chemicals Co. (Qingdao, China). ^1^H-NMR (400 MHz), ^13^C-NMR (100 MHz), and ^31^P-NMR (160 MHz) were performed on JNM-ECA400 (JEOL, Tokyo, Japan), with TMS as an internal standard and 85% H_3_PO_4_ as the external standard, respectively. The high resolution mass spectrum for new compounds was obtained with Agilent TOF G6230A (Agilent Technologies, Santa Clara, CA, USA). The mass spectrum of oligonucleotides was obtained with a HTCS Oligo LC/MS system (Thermo Finnigan, Somerset, NJ, USA).

*5-[2-(Trifluoroacetylamino)ethylaminocarbonylmethyl]-2’-deoxyuridine* (**4b**). To the solution of 1,2-diaminoethane (2.5 mL, 38 mmol) in methanol (2.5 mL) at 60 °C, a solution of compound **4a** (1.14 g, 3.8 mmol) in methanol (5 mL) was slowly added. After stirring for 4 h, the solvent and extra reagents were evaporated off in a vacuum. The residue was dissolved in methanol (10 mL), and triethylamine (2.1 mL) and ethyl trifluoroacetate (5 mL) were added. The reaction mixture was stirred at r.t., until the reaction was finished. By flash chromatography, the product was obtained as colorless foam (1.1 g, 68.3%). *R*_f_ (DCM/methanol, 9:1) 0.32. ^1^H-NMR (400 MHz, DMSO-*d*_6_): δ 2.08 (m, 2 H, C2’-H), 3.05 (s, 2 H, CH_2_), 3.18 (m, 4 H, CH_2_CH_2_), 3.56 (m, 2 H, C5’-H), 3.77 (m, 1 H, C4’-H), 4.22 (m, 1 H, C3’-H), 4.96 (t, *J* = 5.5, C5’-OH), 5.24 (d, *J* = 4.2, C3’-OH), 6.17 (t, *J* = 4.8, C1’-H), 7.72 (s, 1 H, C6-H), 7.96 (m, 1 H, CONH), 9.37 (s, 1 H, CONHCOCF_3_), 11.34 (s, 1 H, 3-NH). ^13^C-NMR (400 MHz, DMSO-*d*_6_): δ 35.3, 39.5, 63.4, 72,4, 85.9, 89.3, 110.5, 116.4, 119.3, 140.4, 152.2, 158.2, 158.6, 165.2, 171.7. HRMS (C_15_H_19_F_3_N_4_O_7_ + H^+^, 425.1279): 425.1278; (C_15_H_19_F_3_N_4_O_7_ + Na^+^, 447.1098): 447.1097.

*5-(2-aminoethylaminocarbonylmethyl)-2’-deoxyuridine* (**4**). Compound **4a** (400 mg, 0.94 mmol) was dissolved in methanol (2 mL) and conc. aq. ammonia (40 mL), and after stirring at r.t. for 4 h, the solution was concentrated for flash chromatography. Compound **4** was obtained as white foam (286 mg, 93%). *R*_f_ (DCM/methanol (with ammonia), 1:1) 0.47. ^1^H-NMR (400 MHz, DMSO-*d*_6_): δ 2.09 (m, 2 H, C2’-H), 2.85 (m, 2 H, CH_2_), 3.10 (s, 2 H, CH_2_), 3.27 (m, 2 H, CH_2_), 3.53 (m, 2 H, C5’-H), 3.78 (m, 1 H, C4’-H), 4.24 (m, 1 H, C3’-H), 5.31 (br, 1 H, C3’-OH), 6.18 (m, 1 H, C1’-H), 7.77 (s, 1 H, C6-H), 8.09 (m, 1 H, NH), 8.55 (br, 1 H, NH). ^13^C-NMR (100 MHz, DMSO-*d*_6_): δ 33.9, 37.1, 61.9, 70.9, 84.5, 87.9, 109.0, 139.0, 150.9, 163.8, 170.8. HRMS (C_13_H_20_N_4_O_6_ + H^+^, 329.1456): 329.1456; (C_13_H_20_N_4_O_6_ + Na^+^, 351.1275): 351.1276.

*5’-O-(4,4’-Dimethoxytrityl)-5-[2-(trifluoroacetylamino)ethylaminocarbonylmethyl]-2’-deoxyuridine* (**4c**). After the co-evaporation of compound **4b** (1.20 g, 2.82 mmol) with dried pyridine (5 mL) twice, the residue was dissolved in dried pyridine (2 mL) and stirred at r.t. To the solution, DMTrCl (1.16 g, 3.38 mmol) was added in portions, with TLC monitoring the process. The reaction was stopped by adding methanol (5 mL), and the solution was evaporated to a small volume for flash chromatography on a neutralized silica gel column with triethylamine. The product was obtained as colorless foam (1.28 g, 62.5%). *R*_f_ (DCM/methanol, 9:1) 0.56. ^1^H-NMR (400 MHz, DMSO-*d*_6_): δ 2.18 (m, 2 H, C2’-H), 2.70 (s, 2 H, CH_2_), 3.17 (m, 6 H, CH_2_CH_2_, C5’-H), 3.73 (s, 6 H, 2 OCH_3_), 3.87 (m, 1 H, C4’-H), 411.25 (m, 1 H, C3’-H), 5.32 (d, *J* = 4.5, C3’-OH), 6.20 (t, *J* = 4.8, C1’-H), 6.86, 7.19–7.40 (2 m, 13 H, arom.H), 7.55 (s, 1 H, C6-H), 7.86 (m, 1 H, CONH), 9.33 (s, 1 H, CONHCOCF_3_), 11.38 (s, 1 H, 3-NH). ^13^C-NMR (400 MHz, DMSO-*d*_6_): δ 33.1, 37.5, 55.0, 63.9, 70.5, 84.0, 85.4, 85.8, 108.8, 113.2, 114.5, 117.3, 123.9, 126.8, 127.7, 127.9, 129.7, 135.3, 135.5, 136.2, 138.3, 144.8, 149.6, 150.4, 158.1, 163.2, 169.4. HRMS (C_36_H_37_F_3_N_4_O_9_ + Na^+^, 749.2405): 749.2405.

*3’-O-(2-Cyanoethyl-N,N-diisopropylaminophosphinoxy)-5’-O-(4,4’-dimethoxytrityl)-5-[2-(trifluoroacetylamino)ethylaminocarbonylmethyl]-2’-deoxyuridine* (**4d**). Compound **4c** (0.5 g, 0.688 mmol) was dissolved in redistilled dichloromethane (10 mL), and *N*,*N*-diisopropylammonium tetrazolide (0.14 g) and 2-cyanoethylbis(diisopropylamino)phosphoramidite (0.10 mL) were added to the solution in sequence. After stirring at r.t. for 30 min, the reactin mixture was diluted with redistilled dichloromethane (10 mL). The solution was washed once with ice-cold 2% aq. NaHCO_3_ and brine. The organic layer was dried with MgSO_4_ and concentrated for flash chromatography on a silica gel column neutralized with 3% triethylamine. The product was obtained as colorless foam (0.42 g, 65.8%). *R*_f_ (DCM/methanol, 20:1) 0.42. ^1^H-NMR (400 MHz, CDCl_3_): δ 1.16 (m, 12 H, 4 CH_3_), 2.36–2.76 (m, 6 H, C2’-H, CH_2_, OCH_2_C*H*_2_CN), 3.24–3.90 (m, 16 H, NCH_2_CH_2_N, C5’-H, 2 OCH_3_, 2 CH, OC*H*_2_CH_2_CN), 4.14 (m, 1 H, C4’-H), 4.70 (m, 1 H, C3’-H), 6.31 (m, C1’-H), 6.49 (1 H, NH), 6.83, 7.19–7.43 (2 m, 13 H, arom.H, C6-H), 7.81 (m, 1 H, CONH), 7.89 (s, 1 H, CONHCOCF_3_). ^13^C-NMR (100 MHz, CDCl_3_): δ 20.2, 22.2, 23.2, 24.6, 34.7, 38.9, 40.5, 43.4, 55.3, 62.4, 62.6, 85.2, 87.0, 108.8, 112.7, 113.4, 113.6, 117.7, 127.5, 128.1, 128.3, 128.5, 130.2, 135.2, 138.8, 144.2, 150.0, 158.2, 158.8, 170.0, 171.3. ^31^P-NMR (160 MHz, CDCl_3_): δ 149.46, 149.64. HRMS (C_45_H_54_F_3_N_6_O_10_P + Na^+^, 949.3483): 949.3483.

*5-[2-(Trifluoroacetylamino)ethylaminocarbonylethyl]-2’-deoxyuridine* (**5b**). As described for **4b**, **5b** was prepared from the reaction of **5a** (1.14 g, 3.8 mmol) and 1,2-diaminoethane (2.5 mL, 38 mmol) and subsequent protection with ethyl trifluoroacetate (5 mL) in methanol. The product was obtained as colorless solid (1.1 g, 68.3%). *R*_f_ (DCM/methanol, 9:1) 0.34. ^1^H-NMR (400 MHz, DMSO-*d*_6_): 2.08 (m, 2 H, CH_2_), 2.26 (m, 2 H, C2’-H), 2.43 (m, 2 H, CH_2_), 3.21 (m, 4 H, CH_2_CH_2_), 3.58 (m, 2 H, C5-H), 3.78 (m, 1 H, C4’-H), 4.25 (m, 1 H, C3’-H), 5.03 (t, 1 H, *J* = 5.1, C5’-OH), 5.26 (d, *J* = 4.2, C3’-OH), 6.18 (t, 1 H, *J* = 6.9, C1’-H), 7.66 (s, 1 H, C6-H), 7.98 (m, 1 H, NH), 9.43 (m, 1 H, NH), 11.32 (s, 1 H, NH). ^13^C-NMR (100 MHz, DMSO-*d*_6_): 23.8, 35.1, 38.3, 62.3, 71.4, 84.8, 88.3, 113.6, 137.5, 151.3, 164.3, 172.6. HRMS (C_16_H_21_F_3_N_4_O_7_ + H^+^, 439.1435): 439.1434; (C_16_H_21_F_3_N_4_O_7_ + Na^+^, 461.1255): 461.1255.

*5-(2-Aminoethylaminocarbonylethyl)-2’-deoxyuridine* (**5**). As described for **4**, compound **5** was prepared from **5b** (500 mg, 1.14 mmol) in conc. aq. ammonia (45 mL) as a colorless solid (368 mg, 94.6%), *R*_f_ (DCM/methanol saturated with ammonia, 1:1) 0.50. ^1^H-NMR (400 MHz, DMSO-*d*_6_): 2.08 (m, 2 H, CH_2_), 2.29 (m, 2 H, C2’-H), 2.45 (m, 2 H, CH_2_), 2.84 (m, 2 H, CH_2_), 3.26 (m, 2 H, CH_2_), 3.58 (m, 2 H, C5-H), 3.78 (m, 1 H, C4’-H), 4.25 (m, 1 H, C3’-H), 5.30 (br, 1 H, C3’-OH), 6.18 (t, *J* = 6.9, 1 H, C1’-H), 7.67 (s, 1 H, C6-H), 8.04 (br, 2 H, NH, NH). ^13^C-NMR (100 MHz, DMSO-*d*_6_): 23.2, 34.6, 37.0, 61.9, 71.0, 84.4, 87.8, 113.1, 137.1, 150.8, 163.8, 172.7. HRMS (C_14_H_22_F_3_N_4_O_6_ + H^+^, 343.1612): 343.1611; (C_14_H_22_F_3_N_4_O_6_ + Na^+^, 365.1432): 365.1433.

*5’-O-(4,4’-Dimethoxytrityl)-5-[2-(trifluoroacetylamino)ethylaminocarbonylethyl]-2’-deoxyuridine* (**5c**). As described for **4c**, compound **5c** was prepared from **5b** (1.20 g, 2.82 mmol) and DMTrCl (1.16 g, 3.38 mmol). The product was purified with flash chromatography as a white foam (1.28 g, 62.5%). *R*_f_ (DCM/methanol, 9:1) 0.58. ^1^H-NMR (400 MHz, DMSO-*d*_6_): 2.09–2.26 (m, 6 H, C2’-H, CH_2_CH_2_), 3.13–3.20 (m, 6 H, C5’-H, CH_2_CH_2_), 3.73 (s, 6 H, 2 CH_3_O), 3.86 (m, 1 H, C4’-H), 4.22 (m, 1 H, C3’-H), 5.32 (d, 1 H, *J* = 4.8, C3’-OH), 6.16 (t, 1 H, *J* = 6.7, C1’-H), 6.89, 71.9–7.41 (m, 14 H, C6-H, arom.H), 7.89 (m, 1 H, NH), 9.40 (m, 1 H, NH), 11.38 (m, 1 H, NH). ^13^C-NMR (100 MHz, DMSO-*d*_6_): 23.8, 38.3, 55.9, 64.9, 71.4, 84.9, 86.3, 86.6, 113.8, 114.2, 127.7, 128.6, 128.8, 130.7, 136.4, 136.5, 137.5, 145.8, 151.2, 157.2, 157.5, 159.1, 164.2, 172.5. HRMS (C_37_H_39_F_3_N_4_O_9_ + Na^+^, 763.2561): 763.2561.

*3’-O-(2-Cyanoethyl-N,N-diisopropylaminophosphinoxy)-5’-O-(4,4’-dimethoxytrityl)-5-[2-(trifluoroacetylamino)ethylaminocarbonylethyl]-2’-deoxyuridine* (**5d**). As described for **4d**, **5d** was prepared from the reaction of **5c** (0.51 g, 0.663 mmol) with 2-cyanoethylbis(diisopropylamino)phosphoramidite (0.10 mL) in the presence of *N*,*N*-diisopropylammonium tetrazolide (0.14 g). The product was obtained as white foam (0.42 g, 67.3%). *R*_f_ (DCM/methanol, 20:1) 0.48. ^1^H-NMR (400 MHz, CDCl_3_): δ (ppm) 1.14 (m, 12 H, 4 CH_3_), 2.04–2.64 (m, 10 H, C2’-H, CH_2_CH_2_, 2 CH, CH_2_CN), 3.27–3.88 (m, 16 H, CH_2_CH_2_, C5’-H, 2 CH_3_O, OCH_2_), 4.11 (m, 1 H, C4’-H), 4.63 (m, 1 H, C3’-H), 6.37 (m, 2 H, C1’-H, NH), 6.83, 7.21–7.41 (m, 14 H, C6-H, arom.H), 7.60 (s, 1 H, NH), 8.22 (br, 1 H, NH), 9.80 (br, 1 H, NH). ^13^C-NMR (100 MHz, CDCl_3_): δ (ppm) 20.7, 23.3, 24.7, 24.8, 24.9, 35.6, 38.8, 40.3, 41.5, 43.4, 43.5, 55.6, 58.3, 58.5, 63.3, 73.8, 73.9, 85.0, 85.2, 85.6, 87.0, 113.6, 113.9, 117.6, 118.1, 127.5, 128.3, 128.5, 130.4, 135.6, 137.5, 144.6, 150.6, 159.0, 164.5, 174.2. ^31^P-NMR (160 MHz, CDCl_3_): δ (ppm) 149.26, 149.35. HRMS (C_46_H_56_F_3_N_6_O_10_P + Na^+^, 963.3640): 963.3642.

*5-[3-(Trifluoroacetylamino)propylaminocarbonylmethyl]-2’-deoxyuridine* (**6a**). As described for the preparation of compound **4b**, **6a** was prepared from the reaction of **4a** (3.42 g, 11.4 mmol) with 1,3-diaminopropane (7.5 mL, 114 mmol) in metanol (22.5 mL) at 60 °C, followed by the addition of triethylamine (6.3 mL) and ethyl trifluoroacetate (15 mL). The product was purified with flash chromatography (3.30 g, 66.1%). *R*_f_ (DCM/methanol, 9:1) 0.34. ^1^H-NMR (400 MHz, DMSO-*d*_6_): δ (ppm) 1.60 (m, 2 H, CH_2_C*H*_2_CH_2_), 2.08 (m, 2 H, C2’-H), 2.98–3.10 (m, 6 H, CH_2_, C*H*_2_CH_2_C*H*_2_), 3.55 (m, 2 H, C5’-H), 3.77 (m, 1 H, C4’-H), 4.22 (m, 1 H, C3’-H), 5.00 (br, 1 H, C5’-OH), 5.27 (br, 1 H, C3’-OH), 6.16 (t, *J* = 5.7 Hz, 1 H, C1’-H), 7.74 (s, 1 H, C6-H), 7.88 (t, *J* = 5.3 Hz, NH), 9.40 (s, 1 H, NH), 11.34 (s, 1 H, 3-NH). ^13^C-NMR (100 MHz, DMSO-*d*_6_): δ (ppm) 29.4, 34.4, 37.3, 38.0, 62.4, 71.4, 84.9, 88.3, 109.7, 139.3, 151.4, 157.0, 157.3, 164.2, 170.4. HRMS (C_16_H_21_F_3_N_4_O_7_ + H^+^, 439.1435): 439.1435; (C_16_H_21_F_3_N_4_O_7_ + Na^+^, 461.1255): 461.1252.

*5-[3-(Trifluoroacetylamino)propylaminocarbonylmethyl]-2’-deoxyuridine* (**6**). As described for compound **4**, compound **6a** (500 mg, 1.14 mmol) was deprotected to obtain compound **6** as a colorless solid (347 mg, 88.9%). *R*_f_ (DCM/methanol saturated with ammonia, 1:1) 0.51. ^1^H-NMR (400 MHz, DMSO-*d*_6_): δ (ppm) 1.42 (m, 2 H, CH2), 2.05 (m, 2 H, C2’-H), 2.48 (m, 2 H, CH2), 3.05 (m, 4 H, 2 CH2), 3.53 (m, 2 H, C5’-H), 3.74 (m, 1 H, C4’-H), 4.20 (m, 1 H, C3’-H), 5.08 (br, 2 H, NH2), 6.15 (t, *J* = 6.9, 1 H, C1’-H), 7.70 (s, 1 H, C6-H), 7.81 (m, 1 H, NH). ^13^C-NMR (100 MHz, DMSO-*d*6): δ (ppm) 33.8, 34.3, 37.3, 62.3, 71.3, 84.9, 88.3, 109.7, 139.1, 151.4, 164.2, 170.1. HRMS (C_14_H_22_N_4_O_6_ + H^+^, 343.1612): 343.1611; (C_13_H_20_N_4_O_6_ + Na^+^, 365.1432): 365.1429.

*5’-O-(4,4-Dimethoxytrityl)-5-[3-(trifluoroacetylamino)propylaminocarbonylmethyl]-2’-deoxyuridine* (**6b**). As described for compound **4c**, **6b** was preparaed from **6a** (1 g, 2.28 mmol) and DMT-Cl (0.94 g, 2.74 mmol) in pyridine (2 mL). The product was obtained as colorless foam by flash chromatography (1.04 g, 61.6%). *R*_f_ (DCM/methanol, 9:1) 0.56. ^1^H-NMR (400 MHz, DMSO-*d*_6_): δ (ppm) 1.57 (m, 2 H, CH_2_C*H*_2_CH_2_), 2.19 (m, 2 H, C2’-H), 2.65 (m, 2 H, C5’-H), 2.98–3.26 (m, 6 H, CH_2_, C*H*_2_CH_2_C*H*_2_), 3.73 (s, 6 H, 2 OCH_3_), 3.88 (m, 1 H, C4’-H), 4.29 (m, 1 H, C3’-H), 5.36 (d, *J* = 4.5 Hz, 1 H, C3’-OH), 6.22 (t, *J* = 6.7 Hz, 1 H, C1’-H), 6.87 (m, 4 H, arom.H), 7.19–7.38 (m, 9 H, arom.H), 7.58 (s, 1 H, C6-H), 7.77 (t, *J* = 5.6 Hz, NH), 9.39 (s, 1 H, NH), 11.41 (s, 1 H, 3-NH). ^13^C-NMR (100 MHz, DMSO-*d*_6_): δ (ppm) 29.4, 34.2, 37.2, 38.0, 56.0, 64.8, 71.5, 84.9, 86.5, 86.8, 110.0, 114.2, 127.7, 128.7, 128.9, 130.7, 136.3, 136.4, 139.1, 145.7, 151.4, 159.1, 164.1, 170.1. HRMS (C_37_H_39_F_3_N_4_O_9_ + Na^+^, 763.2561): 763.2561.

*3’-O-(2-Cyanoethyl-N,N-diisopropylaminophosphinoxy)-5’-O-(4,4-dimethoxytrityl)-5-[3-(trifluoroacetylamino)propylaminocarbonylmethyl]-2’-deoxyuridine* (**6c**). As described for compound **4d**, compound **6c** was prepared from **6b** (0.5 g, 0.675 mmol) and 2-cyanoethylbis(diisopropylamino)phosphoramidte (0.28 mL) in dichloromethane (10 mL), in the presence of *N*,*N*-diisopropylammonium tetrazolide (0.13 g). The product was obtained as a colorless solid (0.43 g, 67.7%). *R*_f_ (DCM/methanol, 20:1) 0.46. ^1^H-NMR (400 MHz, CDCl_3_): δ (ppm) 1.14 (m, 12 H), 1.60 (m, 2 H, CH_2_C*H*_2_CH_2_), 2.34–2.68 (6 H, 2 CH, CH_2_CN, C2’-H), 3.12-3.90 (m, 16 H, C*H*_2_CH_2_C*H*_2_, C5’-H, CH_2_, OCH_2_, 2 OCH_3_), 4.08 (m, 1 H, C4’-H), 4.69 (m, 1 H, C3’-H), 6.35 (m, 2 H, C1’-H, NH), 6.77–7.40 (m, 13 H, arom.H), 7.81 (m, 1 H, C6-H), 8.16 (m, 1 H, NH), 9.85 (br, 1 H, NH). ^13^C-NMR (100 MHz, CDCl_3_): δ (ppm) 20.5, 20.6, 20.7, 23.2, 23.3, 24.7, 24.8, 24.9, 25.0, 29.2, 36.2, 36.3, 36.4, 43.4, 43.5, 43.6, 55.5, 55.6, 58.3, 58.5, 63.0, 63.1, 73.2, 73.4, 85.2, 85.3, 85.5, 86.0, 87.2, 109.4, 113.6, 117.8, 118.1, 127.5, 128.4, 128.6, 130.5, 135.5, 135.6, 139.2, 144.4, 150.5, 157.5, 159.0, 164.4, 164.5, 171.1, 171.2. ^31^P-NMR (160 MHz, CDCl_3_): δ (ppm) 149.41. HRMS (C_46_H_56_F_3_N_6_O_10_P + Na^+^, 963.3640): 963.3642.

*5-[3-(Trifluoroacetylamino)propylaminocarbonylethyl]-2’-deoxyuridine* (**7a**). As described for **4b**, the reaction between **5a** (2.28 g, 7.6 mmol) with 1,3-diaminopropane (5 mL, 76 mmol) followed by protection with ethyl trifluoroacetate (10 mL) offered the compound **7a** as a colorless solid (2.1 g, 61.1%). *R*_f_ (DCM/methanol, 9:1) 0.36. ^1^H-NMR (400 MHz, DMSO-*d*_6_): δ (ppm) 1.59 (m, 2 H, CH_2_C*H*_2_CH_2_), 2.06 (m, 2 H, C2’-H), 2.24, 2.42 (2 t, *J* = 7.42 Hz, 4 H, CH_2_CH_2_), 3.01–3.18 (m, 4 H, C*H*_2_CH_2_C*H*_2_), 3.56 (m, 2 H, C5’-H), 3.76 (m, 1 H, C4’-H), 4.22 (m, 1 H, C3’-H), 5.00 (t, *J* = 5.4, Hz, 1 H, C5’-OH), 5.22 (d, *J* = 4.2 Hz, 1 H, C3’-OH), 6.15 (t, *J* = 7.1 Hz, 1 H, C1’-H), 7.63 (s, 1 H, C6-H), 7.84 (m, 1 H, NH), 9.36 (s, 1 H, NH), 11.29 (s, 1 H, 3-NH). ^13^C-NMR (100 MHz, DMSO-d6): δ (ppm) 22.9, 28.5, 34.1, 36.1, 37.1, 61.4, 70.5, 83.9, 87.4, 112.6, 136.6, 150.4, 163.3, 171.4. HRMS (C_17_H_23_F_3_N_4_O_7_ + H^+^, 453.1592): 453.1592; (C_17_H_23_F_3_N_4_O_7_ + Na^+^, 475.1411): 475.1411.

*5-[3-(Trifluoroacetylamino)propylaminocarbonylethyl]-2’-deoxyuridine* (**7**). As described for compound **4**, compound **7a** (300 mg, 0.66 mmol) was deprotected in conc. aq. ammonia (30 mL). The product was obtained as a colorless solid (229 mg, 97%). *R*_f_ (DCM/methanol saturated with ammonia, 1:1) 0.53. ^1^H-NMR (400 MHz, DMSO-*d*_6_): 1.67 (m, 2 H, CH_2_), 2.08 (m, 2 H, C2’-H), 2.28 (m, 2 H, CH_2_), 2.42 (m, 2 H, CH_2_), 2.78 (m, 2 H, CH_2_), 3.11 (m, 2 H, CH_2_), 3.59 (m, 2 H, C5’-H), 3.78 (m, 1 H, C4’-H), 4.25 (m, 1 H, C3’-H), 5.34 (br, 1 H, C5’-OH), 6.17 (t, *J* = 6.9, 1 H, C1’-H), 7.67 (s, 1 H, C6-H), 8.04 (m, 1 H, NH, NH). ^13^C-NMR (100 MHz, DMSO-*d*_6_): 23.3, 28.0, 34.5, 36.0, 37.2, 61.9, 70.9, 84.4, 87.8, 113.1, 116.1, 119.1, 137.0, 150.8, 158.9, 159.2, 163.8, 172.3. HRMS (C_15_H_24_N_4_O_6_+ H^+^, 357.1769): 357.1769; C_15_H_24_N_4_O_6_ + Na^+^, 379.1588): 379.1582.

*5’-O-(4,4-Dimethoxytrityl)-5-[3-(trifluoroacetylamino)propylaminocarbonylethyl]-2’-deoxyuridine* (**7b**). As described for **4c**, compound **7a** (1 g, 2.21 mmol) was reacted with DMTrCl (0.84 g, 2.65 mmol) in dried pyridine (2 mL) to obtain the product as a colorless solid (1.18 g, 70.7%). *R*_f_ (DCM/methanol, 9:1) 0.55. ^1^H-NMR (400 MHz, DMSO-*d*_6_): δ (ppm) 1.57 (m, 2 H, CH_2_C*H*_2_CH_2_), 2.12—2.25 (m, 6 H, C2’-H, CH_2_CH_2_), 2.98–3.18 (m, 6 H, C5’-H, C*H*_2_CH_2_C*H*_2_), 3.73 (s, 6 H, 2 OCH_3_), 3.86 (m, 1 H, C4’-H), 4.22 (m, 1 H, C3’-H), 5.32 (d, *J* = 4.5 Hz, 1 H, C3’-OH), 6.16 (t, *J* = 6.8 Hz, 1 H, C1’-H), 6.88 (m, 4 H, arom.H), 7.19–7.41 (m, 10 H, C6-H, arom.H), 7.76 (m, 1 H, NH), 9.38 (s, 1 H, NH), 11.39 (s, 1 H, 3-NH). ^13^C-NMR (100 MHz, DMSO-*d*_6_): δ (ppm) 23.8, 29.4, 35.1, 36.9, 38.0, 55.9, 64.8, 71.3, 84.9, 86.3, 86.6, 113.8, 114.1, 127.6, 128.6, 128.8, 130.6, 136.3, 136.4, 137.3, 145.7, 151.2, 159.0, 164.1, 172.1. HRMS (C_38_H_41_F_3_N_4_O_9_ + H^+^, 754.2826): 754.2826; (C_38_H_41_F_3_N_4_O_9_ + Na^+^, 777.2713): 777.2713.

*3’-O-(2-Cyanoethyl-N,N-diisopropylaminophosphinoxy)-5’-O-(4,4-dimethoxytrityl)-5-[3-(trifluoroacetylamino)propylaminocarbonylmethyl]-2’-deoxyuridine* (**7c**). As described for compound **4d**, **7c** was prepared from **7b** (0.5 g, 0.66 mmol) with 2-cyanoethylbis(diisopropylamino)phosphoramidite (0.17 mL) in dichloromethane (10 mL), in the presence of *N*,*N*-diisopropylammonium tetrazolide (0.14 g). The product was obtained as white foam (0.45 g, 71%). *R*_f_ (DCM/methanol, 20:1) 0.39. ^1^H-NMR (400 MHz, DMSO-*d*_6_): δ (ppm) 1.02–1.51 (m, 14 H, 2 NCH(C*H*_3_)_2_, CH_2_C*H*_2_CH_2_), 2.04–2.77 (m, 8 H, C2’-H, C*H*_2_C*H*_2_, OCH_2_C*H*_2_CN), 3.12–3.86 (m, 14 H, 2 NC*H*(CH_3_)_2_, C*H*_2_CH_2_C*H*_2_, 2 CH_3_O, OC*H*_2_CH_2_CN), 4.18 (m, 2 H, C5’-H), 4.61 (m, 1 H, C4’-H), 5.76 (m, 1 H, C3’-H), 6.36 (m, 1 H, C1’-H), 6.84, 7.29 (2 m, 14 H, NH, arom.H), 8.16 (s, 1 H, C6-H), 9.12 (br, 1 H, NH), 11.15 (br, 1 H, NH). ^13^C-NMR (100 MHz, CDCl_3_): δ (ppm) 23.6, 24.1 24.9, 29.4, 36.0, 40.4, 43.8, 55.7, 58.4, 63.7, 85.2, 87.1, 113.7, 127.6, 128.5, 130.6, 136.1, 137.4, 145.0, 150.4, 159.2, 173.7. ^31^P-NMR (160 MHz, CDCl_3_): δ (ppm) 149.26, 149.40. HRMS (C_47_H_58_F_3_N_6_O_10_P + Na^+^, 977.3796): 977.3795.

### 3.2. T_m_ Measurement

The melting temperatures of the DNAzyme-substrate complexes were determined using a Varian Bio 100 spectrometer (Varian, Palo Alto, CA, USA). An equal amount (2.0 µM) of DNAzyme and the full-DNA substrate were mixed in a buffer containing 50 mM Tris-HCl pH 7.4 and 2 mM Mg^2+^. The solution was incubated at 85 °C for 10 min. Subsequently, it was cooled slowly to 10 °C, at a rate of 1 °C/min. The absorbance at 260 nm was measured over the temperature range. The first derivative of the melting curve corresponds to the melting temperature (T_m_) of the DNAzyme-substrate complex. T_m_ was given as the mean value of the two measurements, with a variation of <0.5 °C.

### 3.3. Circular Dichroism

The samples (2.0 µM in a buffer of 50 mM Tris-HCl (pH 7.4) and 2 mM Mg^2+^) in 1 cm cuvettes were used for CD spectra. Data were collected on a Jasco J810 spectropolarimeter (JASCO Corporation, Tokyo, Japan) from 350 nm to 200 nm, with a scan speed of 100 nm/min, at 2 nm intervals. Each sample was scanned three times and smoothed with the Jasco smoothing algorithm.

### 3.4. Cleavage Reactions under Single-Turnover Conditions

The substrate was radio-labeled with 50 µCi of [γ-^32^P]ATP using 10 U of T4 polynucleotide kinase (Takara, Dalian, China). After incubation at 37 °C for 1 h, the radioactive substrate was extracted with a Sep-Pak column (Waters Corporation, Milford, MA, USA) and washed with sterilized bidistilled water. The product was eluted from the column with methanol/water (70/30, *v*/*v*), lyophilized, and stored at −30 °C.

The DNAzyme cleavage reaction was conducted under single-turnover conditions, with 2 µM of the DNAzyme and 20 nM of the radio-labeled substrate. Prior to the reaction, the DNAzyme and the substrate were mixed in the buffer of 50 mM Tris-HCl, and the solution was heated at 75 °C for 10 min. After cooling to 37 °C, the buffer containing Mg^2+^ was added to initiate the reaction, with the final concentration of 2.18 mM Mg^2+^. Aliquots were taken at defined intervals and mixed with stopping buffer (8 M urea and EDTA) immediately. These samples were analyzed with a 20% denaturing polyacrylamide gel, the substrate and the product were separated, and their radioactivity were recorded with a Phosphoimager (Cyclone Plus Phosphor Scanning System Molders C431200, PerkinElmer, Downer Crove, IL, USA) as the percentage of product after different time intervals. The cleavage reaction of each DNAzyme was repeated at least three times on different days, and less than 15% variation was permitted. The results were reported as the average values of at least three measurements.

## 4. Conclusions

In the complex tertiary structure of the catalytic core of 10-23 DNAzyme, the functional groups around each nucleobase are supposed to play an active role in forming the catalytic conformation, in addition to the base stacking interaction. With the 2’-deoxythymidine analogues **1**–**7** at T4 or T8, the hydroxyl, imidazolyl, and amino groups were introduced, and modifications on the functional group of the nucleobases were conducted. Both negative and positive effects on the catalytic activity of DNAzyme were obtained, which indicated that more interactions were induced by these new external functional groups. These results implied that the 5-methyl group of the most conserved T4 and the least conserved T8 are supposed to be in contact with other residues in the catalytic conformation. This kind of contact could be modulated by other functional groups. T8 is more compatible with the 5-modifications than T4, but a positive effect could be expected at both residues by a delicate 5-substituent, although it is still far from a rational design. On the other hand, the conservation of T4 and T8 could be recognized at the level of the nucleobase, but might be more accurate at the level of the functional group.

In the complex tertiary structure of 10-23 DNAzyme, chemical modification at the level of functional groups from either the nucleobase or sugar-phosphate is the first step for the optimization approach. Nucleoside analogues of the corresponding canonical residues could be used for this purpose. Here, we learnt that the 5-position of T4 and T8 could be modified with the external hydroxyl and amino groups. This kind of chemical modification presents a new start for a better catalytic ability, and furthermore, the modified catalytic core could offer a stronger resistance to endonucleases, which is helpful for the therapeutic applications of 10-23 DNAzyme. Further delicate design of 5-substitutents of 2’-deoxythymine is in progress for more efficient DNAzymes.

## Figures and Tables

**Table 1:** Characterization results of DNAzymes and substrates with molecular mass spectrum measurements.

DNAzyme	Sequence	MS (Calc.)	MS (Found)
**DZ01**	5’-d(tgc tct cca GGC TAG CTA CAA CGA cct gca cct)-3’	9994.4	9992.5
**DZ-T4-1**	5’-d(tgc tct cca GGC **1**AG CTA CAA CGA cct gca cct)-3’	10010.5	10015.1
**DZ-T8-1**	5’-d(tgc tct cca GGC TAG C**1**A CAA CGA cct gca cct)-3’	10010.5	10014.0
**DZ-T4-2**	5’-d(tgc tct cca GGC **2**AG CTA CAA CGA cct gca cct)-3’	10038.5	10038.5
**DZ-T8-2**	5’-d(tgc tct cca GGC TAG C**2**A CAA CGA cct gca cct)-3’	10038.5	10038.3
**DZ-T4-3**	5’-d(tgc tct cca GGC **3**AG CTA CAA CGA cct gca cct)-3’	10072.5	10072.8
**DZ-T8-3**	5’-d(tgc tct cca GGC TAG C**3**A CAA CGA cct gca cct)-3’	10072.5	10074.6
**DZ-T4-4**	5’-d(tgc tct cca GGC **4**AG CTA CAA CGA cct gca cct)-3’	10080.53	10079.3
**DZ-T8-4**	5’-d(tgc tct cca GGC TAG C**4**A CAA CGA cct gca cct)-3’	10080.53	10078.5
**DZ-T4-T8-4**	5’-d(tgc tct cca GGC **4**AG C**4**A CAA CGA cct gca cct)-3’	10166.62	10164.8
**DZ-C13-4**	5’-d(tgc tct cca GGC TAG CTA **4**AA CGA cct gca cct)-3’	10094.55	10093.9
**DZ-T4-5**	5’-d(tgc tct cca GGC **5**AG CTA CAA CGA cct gca cct)-3’	10094.6	10093.4
**DZ-T8-5**	5’-d(tgc tct cca GGC TAG C**5**A CAA CGA cct gca cct)-3’	10094.6	10094.0
**DZ-T4-T8-5**	5’-d(tgc tct cca GGC **5**AG C**5**A CAA CGA cct gca cct)-3’	10194.7	10196.1
**DZ-C13-5**	5’-d(tgc tct cca GGC TAG CTA **5**AA CGA cct gca cct)-3’	10109.6	10113.0
**DZ-T4-6**	5’-d(tgc tct cca GGC **6**AG CTA CAA CGA cct gca cct)-3’	10094.55	10092.9
**DZ-T8-6**	5’-d(tgc tct cca GGC TAG C**6**A CAA CGA cct gca cct)-3’	10094.55	10093.3
**DZ-T4-T8-6**	5’-d(tgc tct cca GGC **6**AG C**6**A CAA CGA cct gca cct)-3’	10194.67	10193.4
**DZ-C13-6**	5’-d(tgc tct cca GGC TAG CTA **6**AA CGA cct gca cct)-3’	10109.6	10109.5
**DZ-T4-7**	5’-d(tgc tct cca GGC **7**AG CTA CAA CGA cct gca cct)-3’	10108.7	10110.6
**DZ-T8-7**	5’-d(tgc tct cca GGC TAG C**7**A CAA CGA cct gca cct)-3’	10108.7	10115.6
**DZ-T4-T8-7**	5’-d(tgc tct cca GGC **7**AG C**7**A CAA CGA cct gca cct)-3’	10222.8	10223.1
**DZ-C13-7**	5’-d(tgc tct cca GGC TAG CTA **7**AA CGA cct gca cct)-3’	10123.7	10124.9
**D19**	5’-d(AGG TGC AGG ATG GAG AGC A)-3’	5966.9	5966.4
**D19AU**	5’-d(AGG TGC AGG)-r AU-d(TG GAG AGC A)-3’	5984.9	5983.0

**Table 2:** Thermal stabilities of the DNAzyme-substrate complexes under the catalytic reaction conditions.

DNAzyme	T_m_ (°C)	DNAzyme	T_m_ (°C)	DNAzyme	T_m_ (°C)
**DZ01**	51.0				
**DZ-T4-1**	51.2	**DZ-T8-1**	50.1	**DZ-T4-T8-4**	50.3
**DZ-T4-2**	50.9	**DZ-T8-2**	49.9	**DZ-T4-T8-5**	49.7
**DZ-T4-3**	49.2	**DZ-T8-3**	49.9	**DZ-T4-T8-6**	50.1
**DZ-T4-4**	50.8	**DZ-T8-4**	50.3	**DZ-T4-T8-7**	50.5
**DZ-T4-5**	49.9	**DZ-T** **8-5**	50.3	**DZ-C13-4**	50.6
**DZ-T4-6**	50.5	**DZ-T8-6**	50.2	**DZ-C13-5**	51.3
**DZ-T4-7**	49.0	**DZ-T8-7**	49.3	**DZ-C13-6**	51.3
				**DZ-C13-7**	49.7

Three measurements were conducted for each T_m_, with a standard error of 0.5 °C.

**Table 3:** The observed rate constants of DNAzymes under single-turnover conditions.

DNAzyme	*k*_obs_ (min^−1^)	DNAzyme	*k*_obs_ (min^−1^)
**DZ01**	0.0051 ± 0.0005		
**DZ-T4-1**	0.0060 ± 0.0005	**DZ-T8-1**	0.0076 ± 0.0005
**DZ-T4-2**	0.0032 ± 0.0001	**DZ-T8-2**	0.0064 ± 0.0003
**DZ-T4-3**	0.0009 ± 0.00006	**DZ-T8-3**	0.0022 ± 0.00006
**DZ-T4-4**	0.0034 ± 0.0003	**DZ-T8-4**	0.0036 ± 0.0005
**DZ-T4-5**	0.0035 ± 0.0004	**DZ-T8-5**	0.0088 ± 0.0005
**DZ-T4-6**	0.0037 ± 0.0003	**DZ-T8-6**	0.0054 ± 0.0008
**DZ-T4-7**	0.0026 ± 0.0004	**DZ-T8-7**	0.0072 ± 0.0008
**DZ-T4-T8-4**	0.0062 ± 0.0006	**DZ-C13-4**	-
**DZ-T4-T8-5**	0.0064 ± 0.0006	**DZ-C13-5**	-
**DZ-T4-T8-6**	0.0052 ± 0.0007	**DZ-C13-6**	-
**DZ-T4-T8-7**	0.0059 ± 0.0005	**DZ-C13-7**	-

## Supplementary Materials

The mass spectrum of oligonucleotides is available online.
