# Supplementary material for: Studies on the Two Thymine Residues in the Catalytic Core of 10-23 DNAzyme: The Impact on the Catalysis of Their 5-Substituted Functional Groups
Source: Molecules. 2017 Jun 22;22(7):1011. doi: 10.3390/molecules22071011 (PMC6152017; doi:10.3390/molecules22071011)
Supplement: Supplementary file 1 [file molecules-22-01011-s001.pdf]

Supplementary Materials

Studies on the Two Thymine Residues in the Catalytic Core of 10-23 DNAzyme: the Impact on Catalysis of Their 5-Substituted Functional Groups

Pengyu Li, ShanShan Du, Yang Li, Junlin He

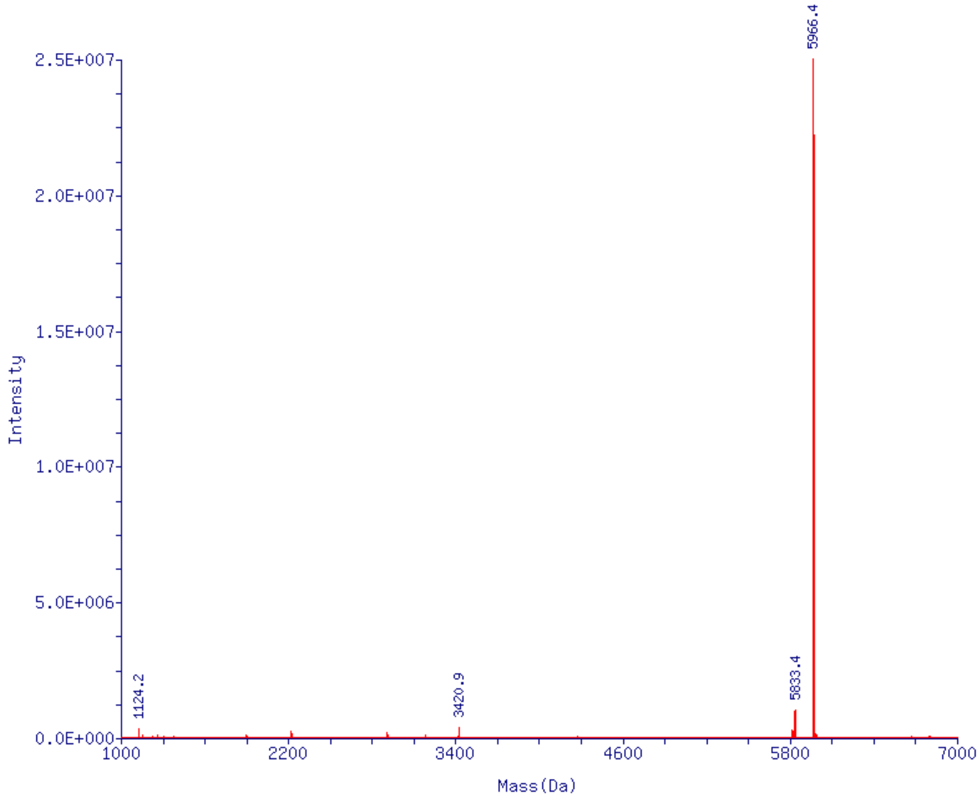

D19

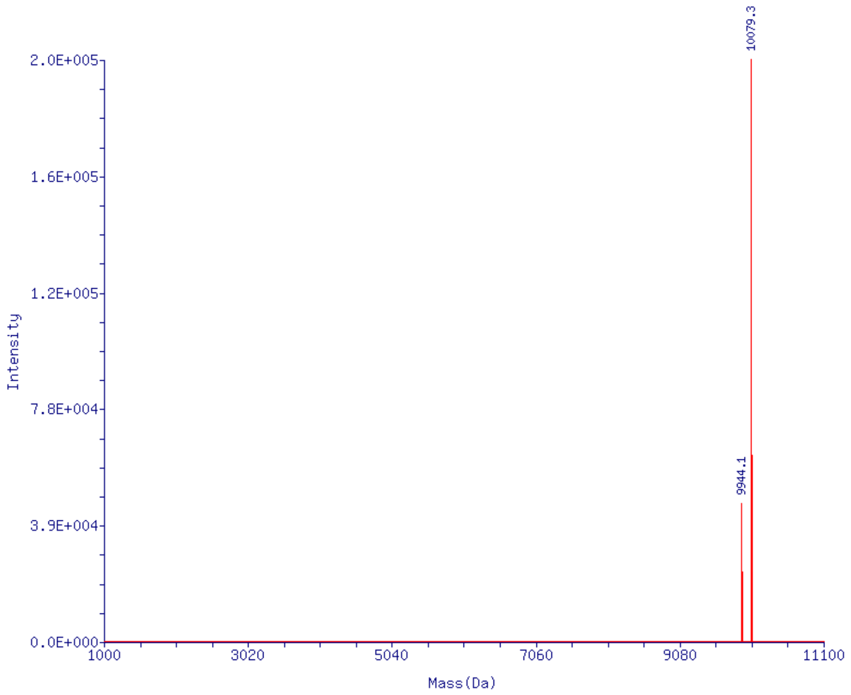

10079.3
9944.1
2.0E+005
1.6E+005
1.2E+005
7.8E+004
3.9E+004
0.0E+000
Intensity
1000
3020
5040
7060
9080
11100
Mass(Da)

DZ-T4-4

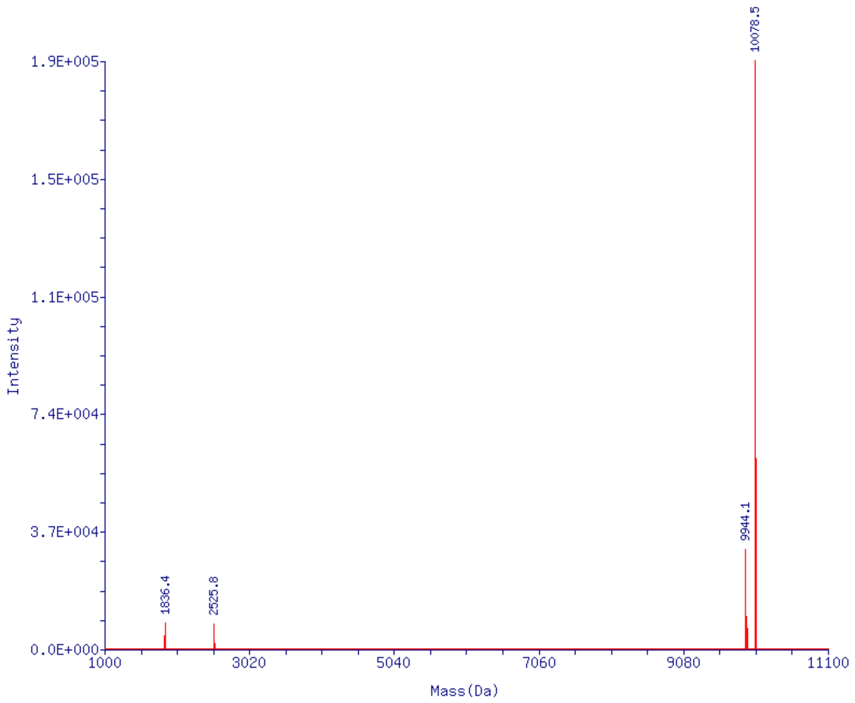

10078.5
9944.1
1836.4
2525.8
1.9E+005
1.5E+005
1.1E+005
7.4E+004
3.7E+004
0.0E+000
Intensity
1000
3020
5040
7060
9080
11100
Mass(Da)

DZ-T8-4

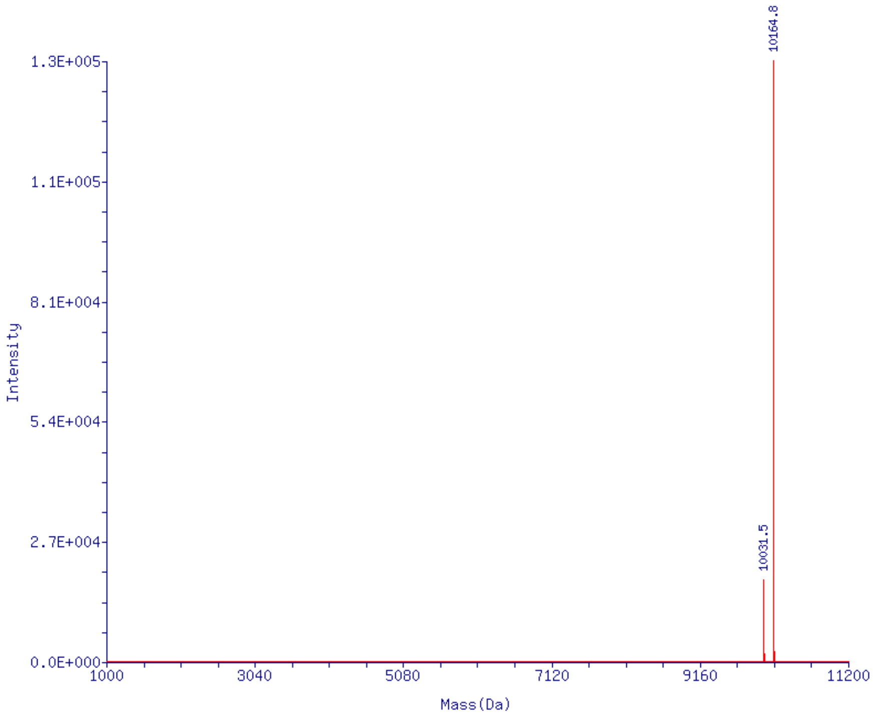

DZ-T4-T8-4

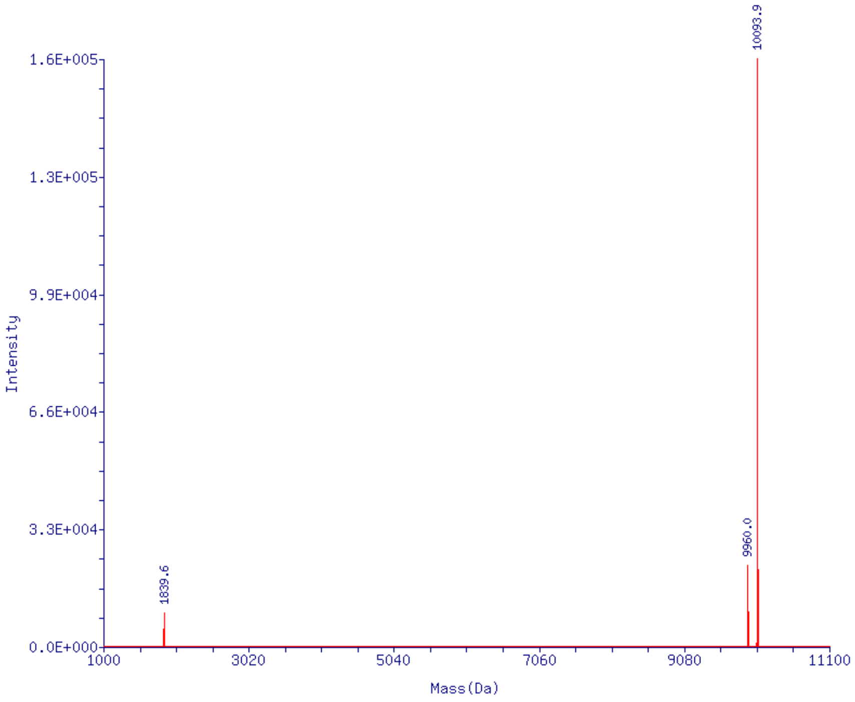

DZ-C13-4

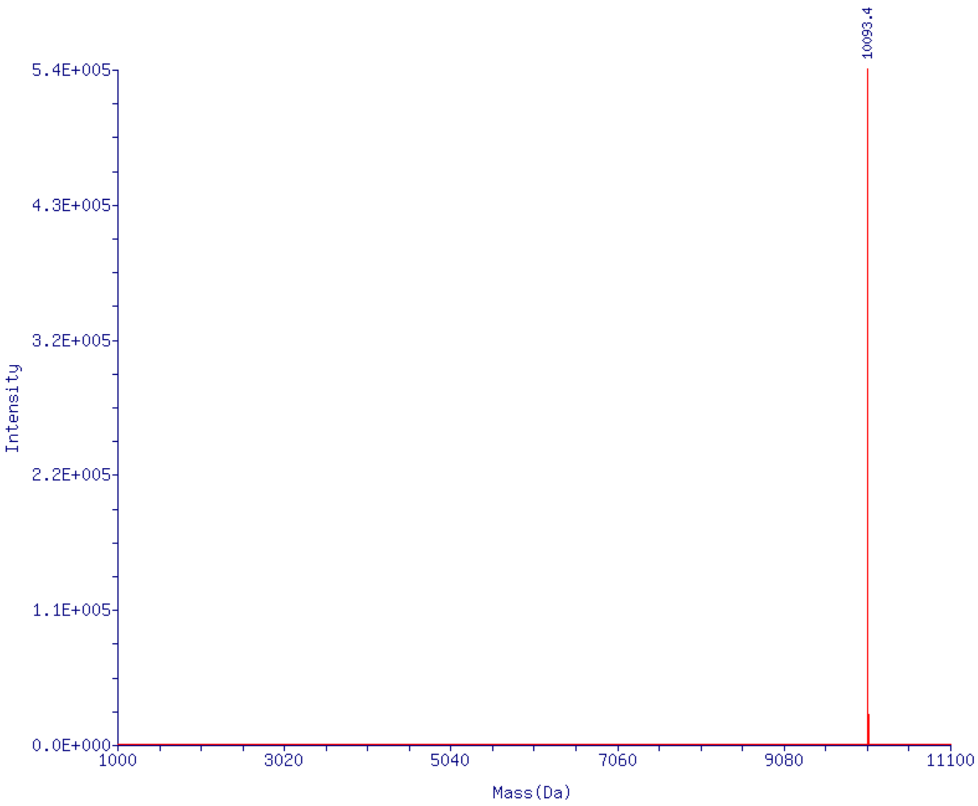
10093.4
5.4E+005
4.3E+005
3.2E+005
2.2E+005
1.1E+005
0.0E+000
Intensity
1000
3020
5040
7060
9080
11100
Mass(Da)

DZ-T4-5

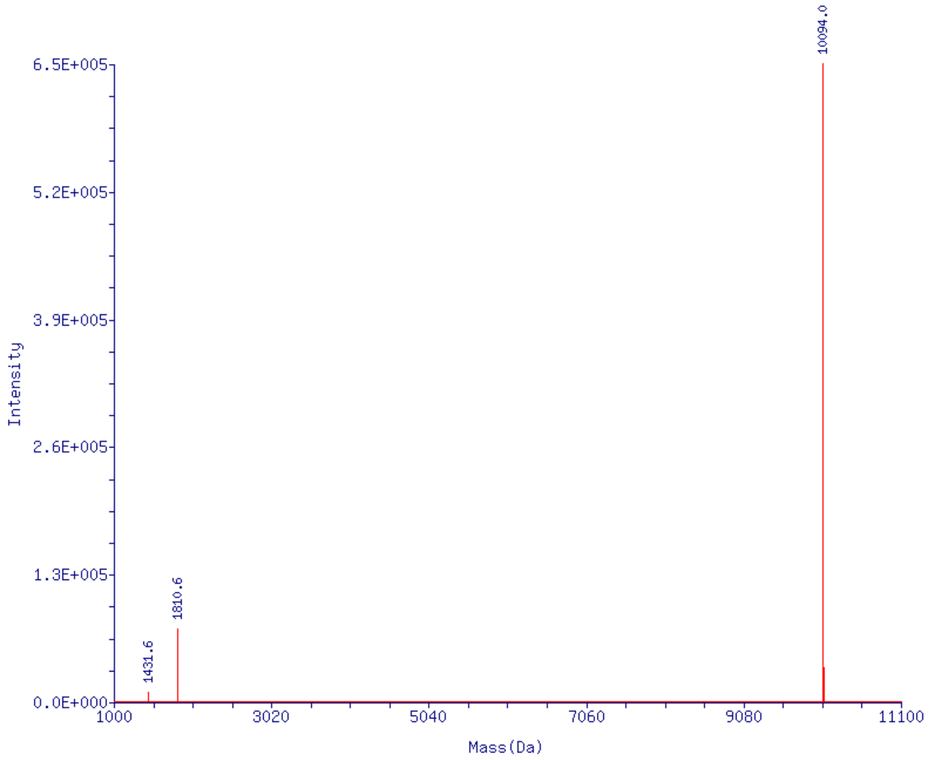
10094.0
6.5E+005
5.2E+005
3.9E+005
2.6E+005
1.3E+005
0.0E+000
Intensity
1810.6
1431.6
1000
3020
5040
7060
9080
11100
Mass(Da)

DZ-T8-5

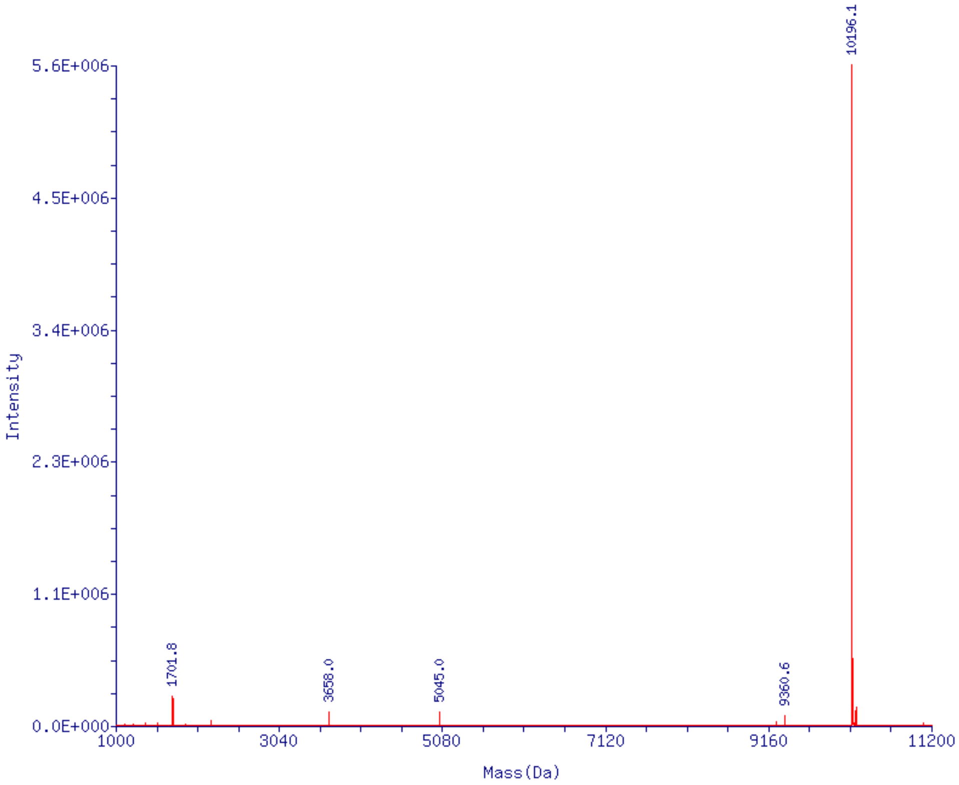

DZ-T4-T8-5

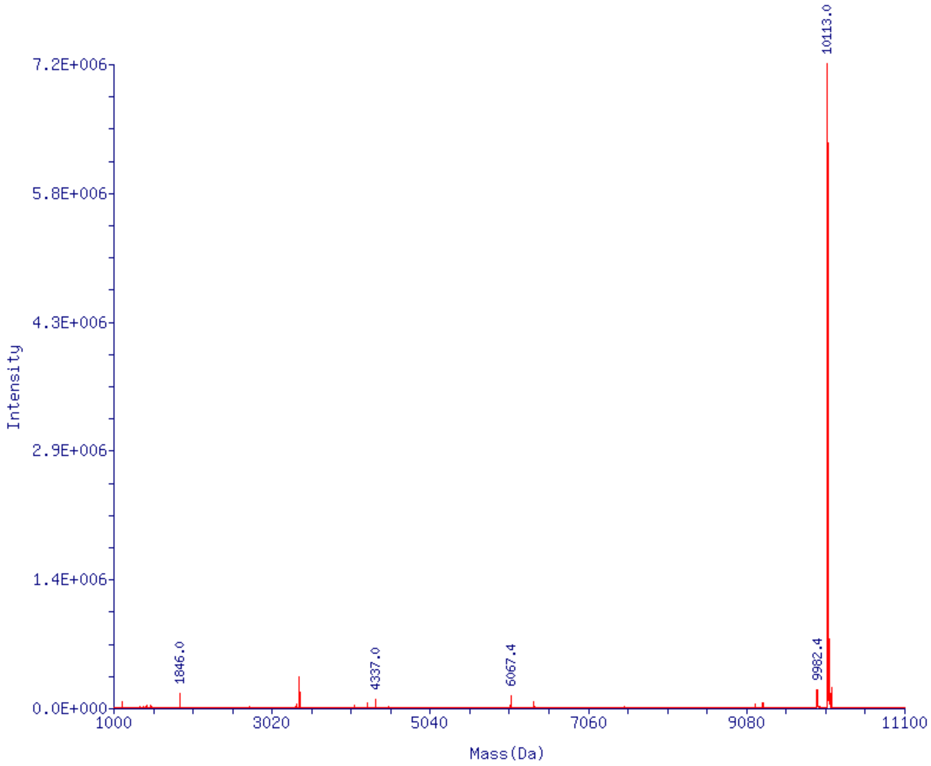

DZ-C13-5

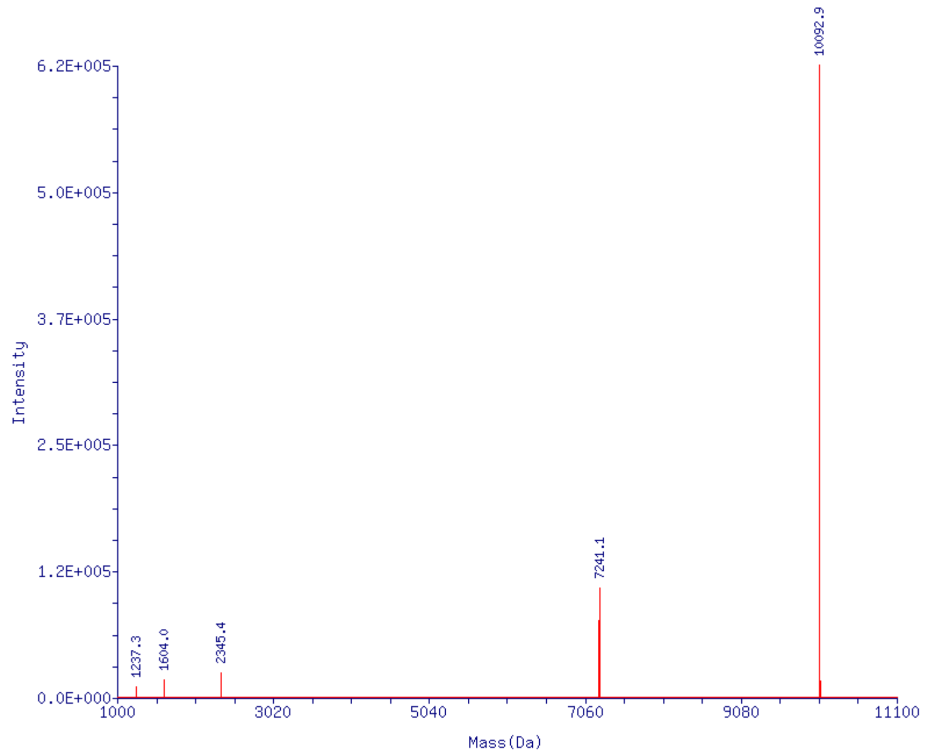

HJLP01 DZ-T4-6

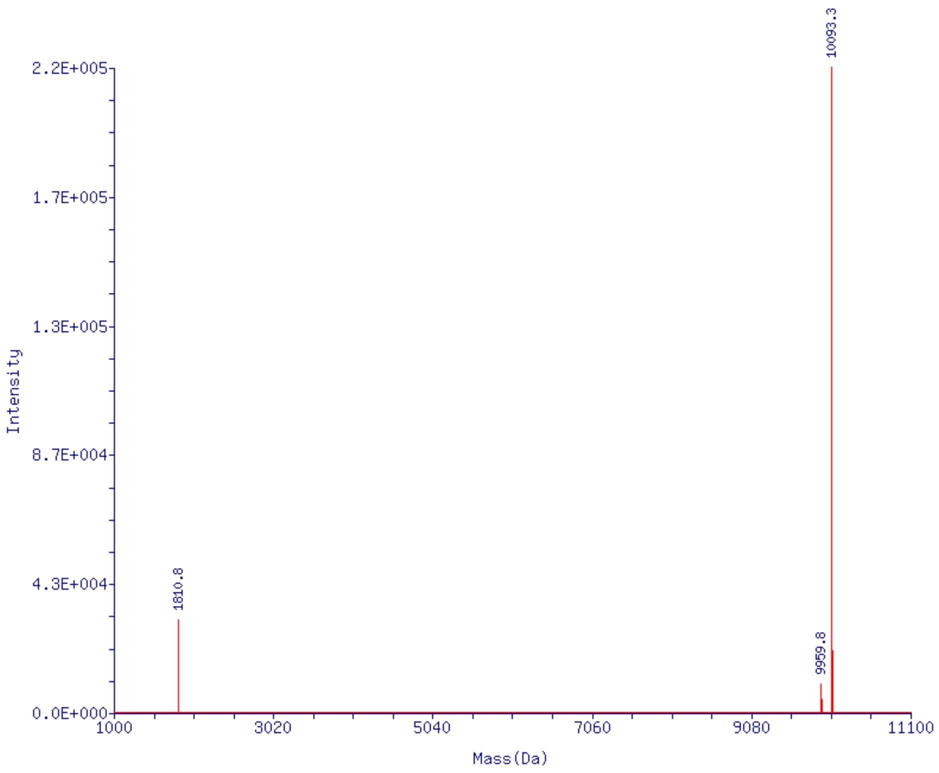

DZ-T8-6

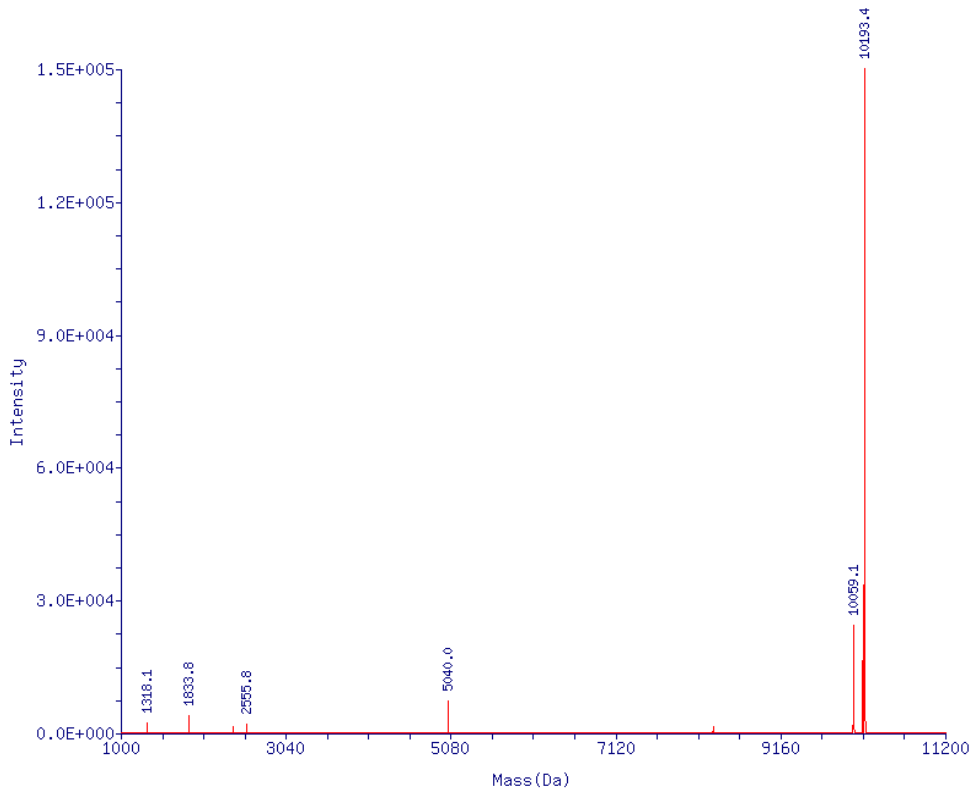

DZ-T4-T8-6

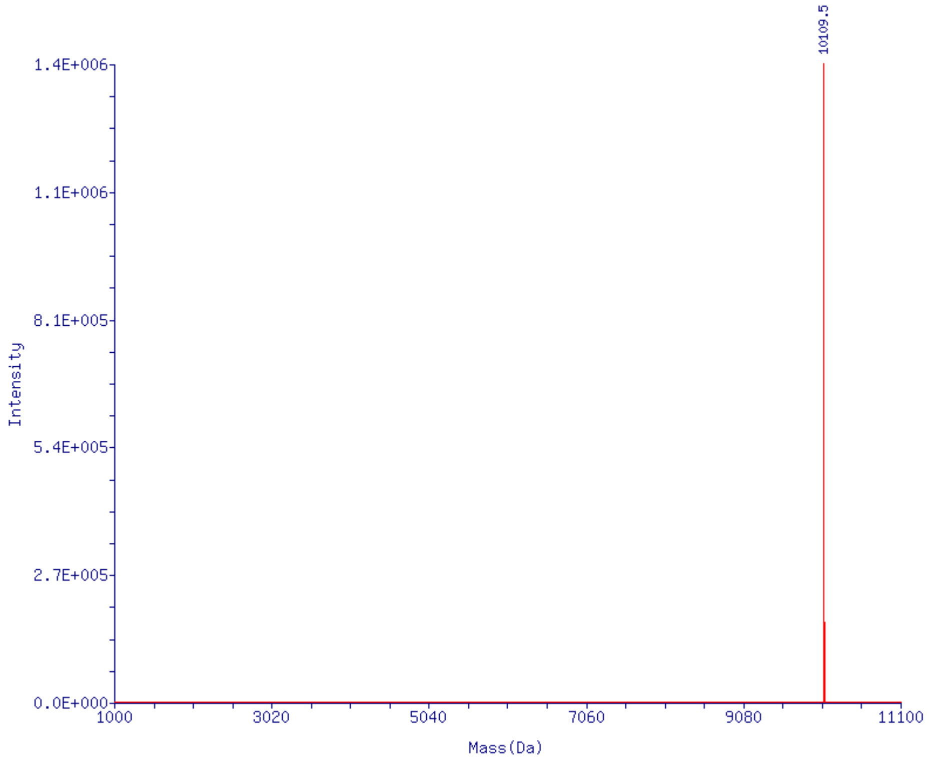

DZ-C13-6

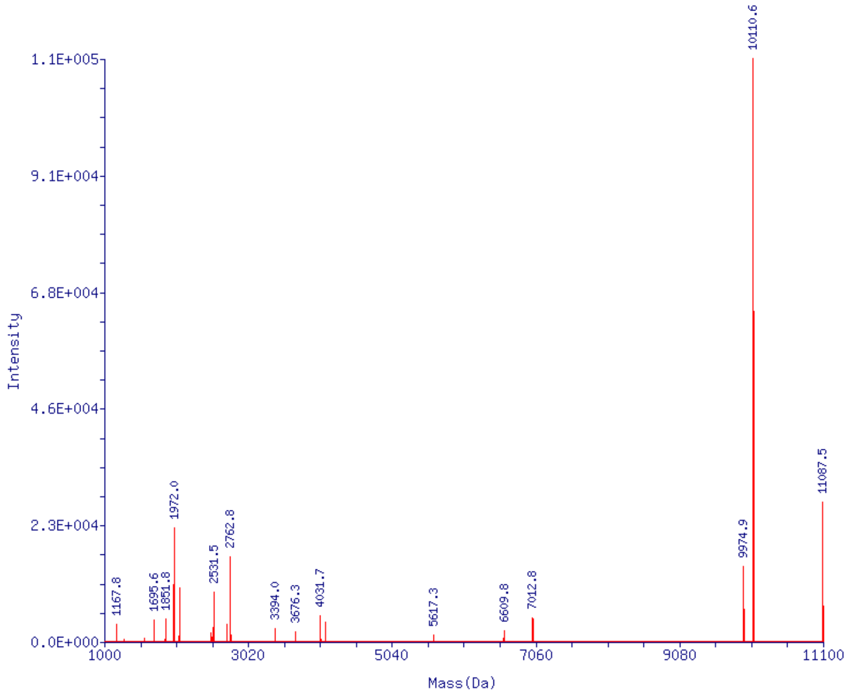
10110.6
1.1E+005
9.1E+004
6.8E+004
4.6E+004
2.3E+004
0.0E+000
Intensity
11087.5
1972.0
2762.8
9974.9
2531.5
1167.8
1695.6
1851.8
3394.0
3676.3
4031.7
5617.3
6609.8
7012.8
1000
3020
5040
7060
9080
11100
Mass(Da)

DZ-T4-7

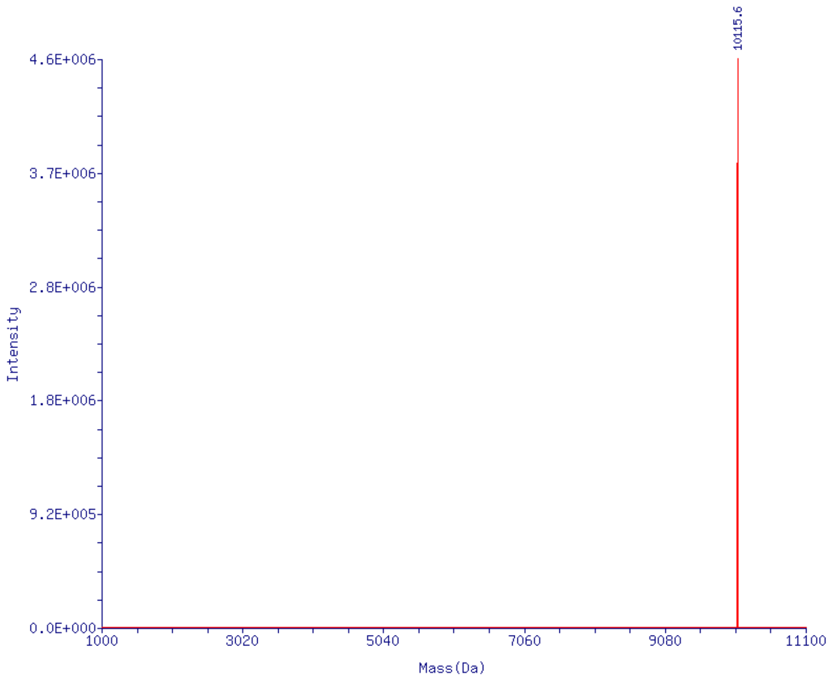
10115.6
4.6E+006
3.7E+006
2.8E+006
1.8E+006
9.2E+005
0.0E+000
Intensity
1000
3020
5040
7060
9080
11100
Mass(Da)

DZ-T8-7

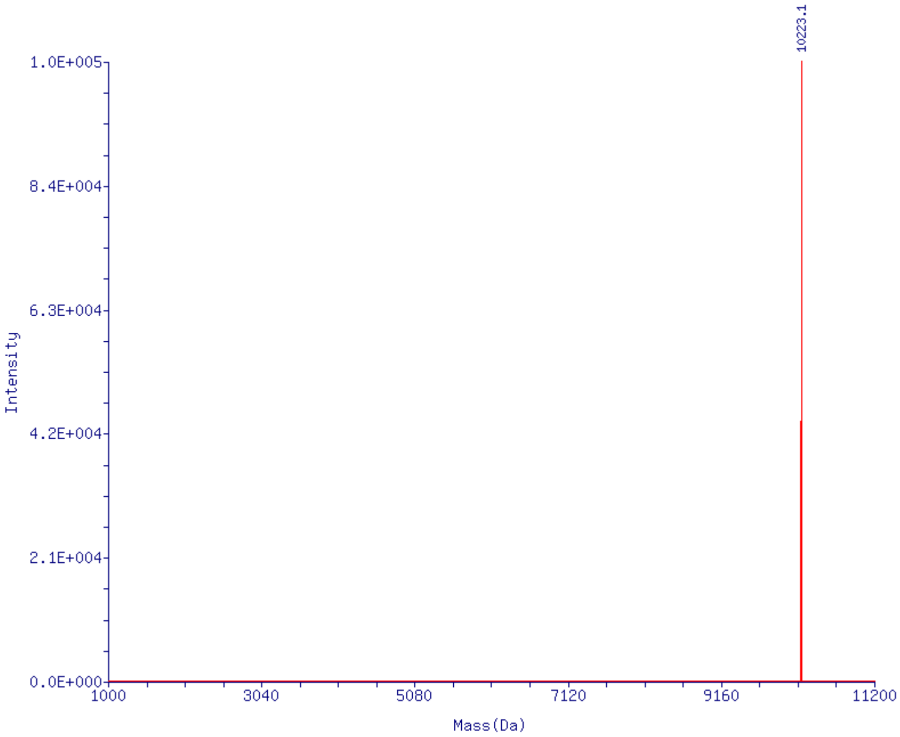

DZ-T4-T8-7

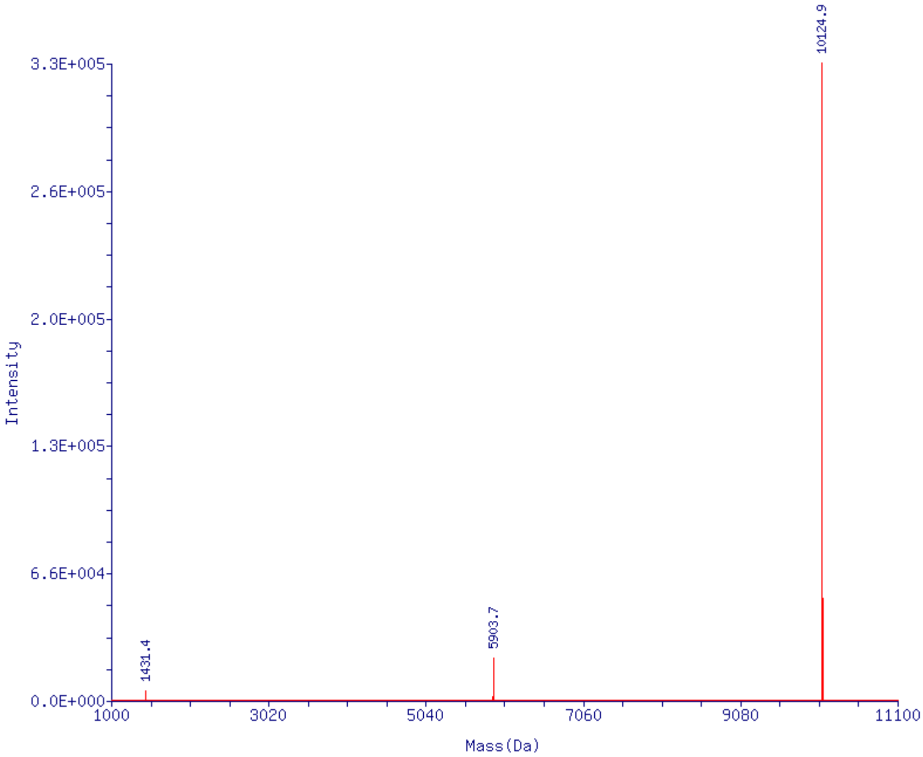

DZ-C13-7
